# Supplementary material for: Spatial Transcriptomics and Single-Nucleus Multi-Omics Analysis Revealing the Impact of High Maternal Folic Acid Supplementation on Offspring Brain Development
Source: Nutrients. 2024 Nov 7;16(22):3820. doi: 10.3390/nu16223820 (PMC11597041; doi:10.3390/nu16223820)
Supplement: Supplementary file 1 [file nutrients-16-03820-s001.zip › nutrients-3281011-Supplementary figures.pdf]

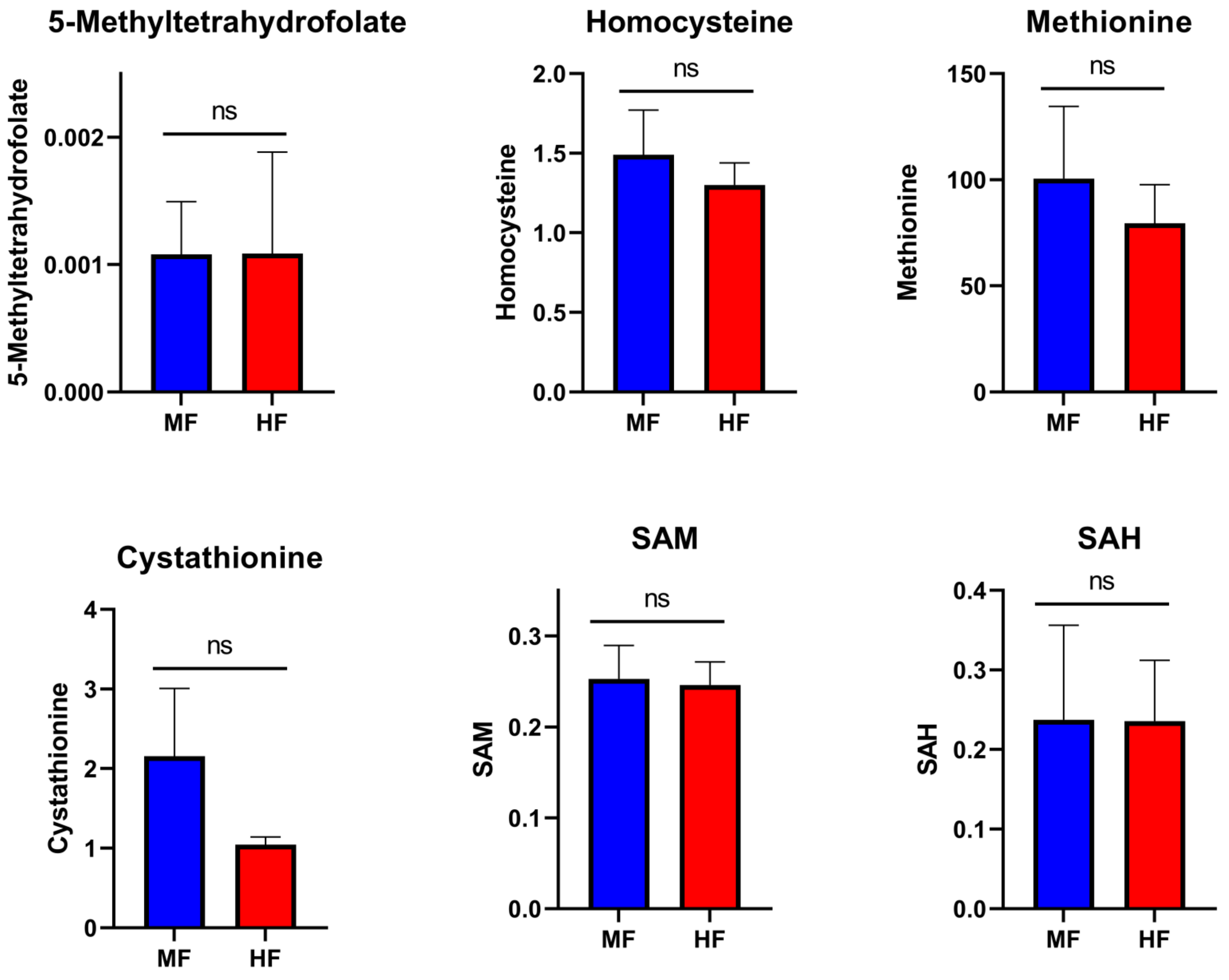

Supplementary Figure S1. Quantification of one−carbon metabolites by LC−MS. Maternal plasma samples were collected when the pups reached P21 and analyzed using LC−MS to quantify one−carbon metabolites: Methyltetrahydrofolate (5−Me−THF), homocysteine (Hcy), methionine (MET), cystathionine (CYSTA), S−adenosylmethionine (SAM), and S−adenosyl−L−homocysteine (SAH). ns. not significant.

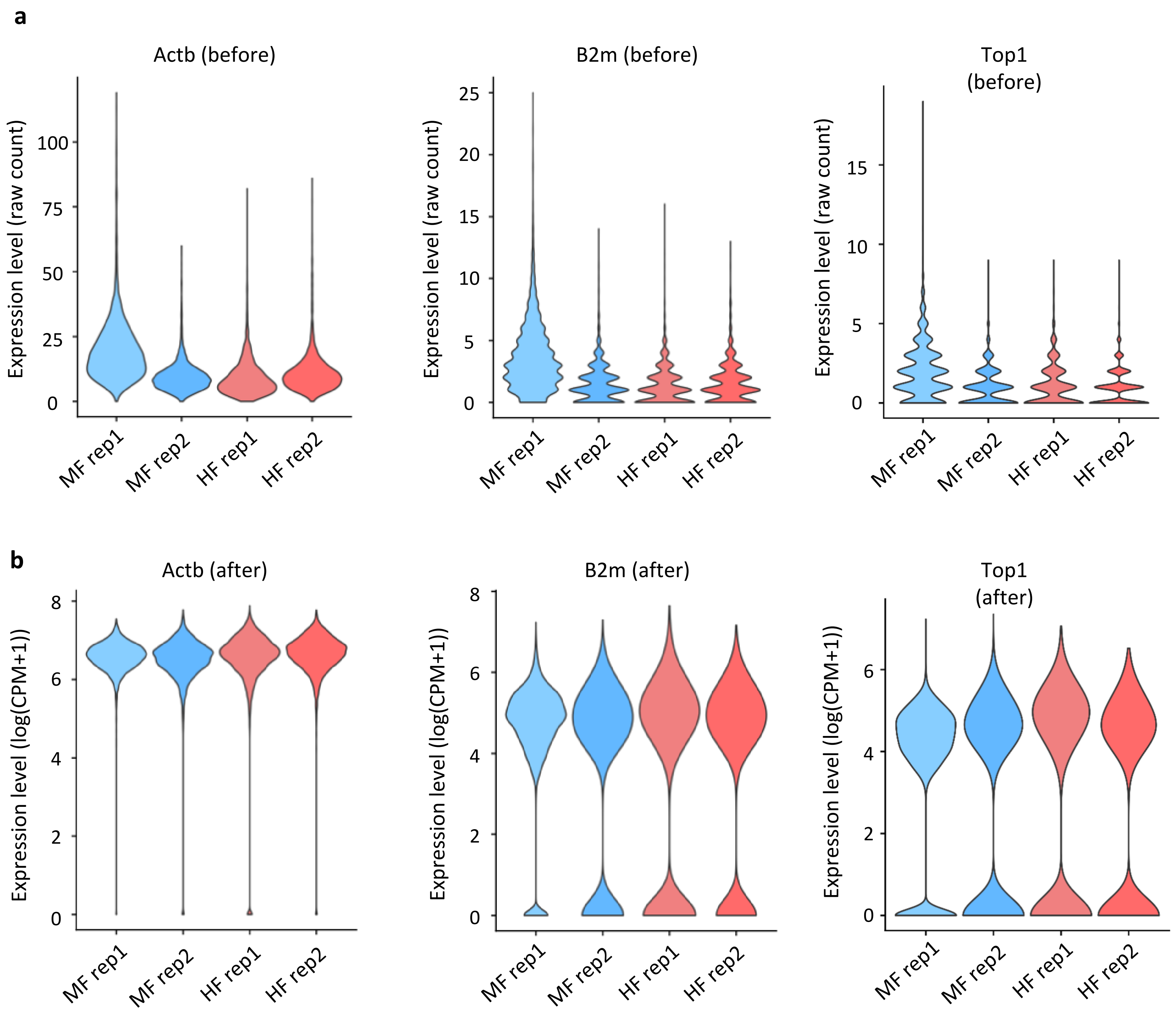

Supplementary Figure S2. Normalization of Visium spatial transcriptomics datasets. a. Violin plot showing the raw counts of the house keeping genes Actb, B2m and Top1 before data normalization. **b**. Violin plot showing the normalized and log transformed counts of Actb, B2m and Top1.

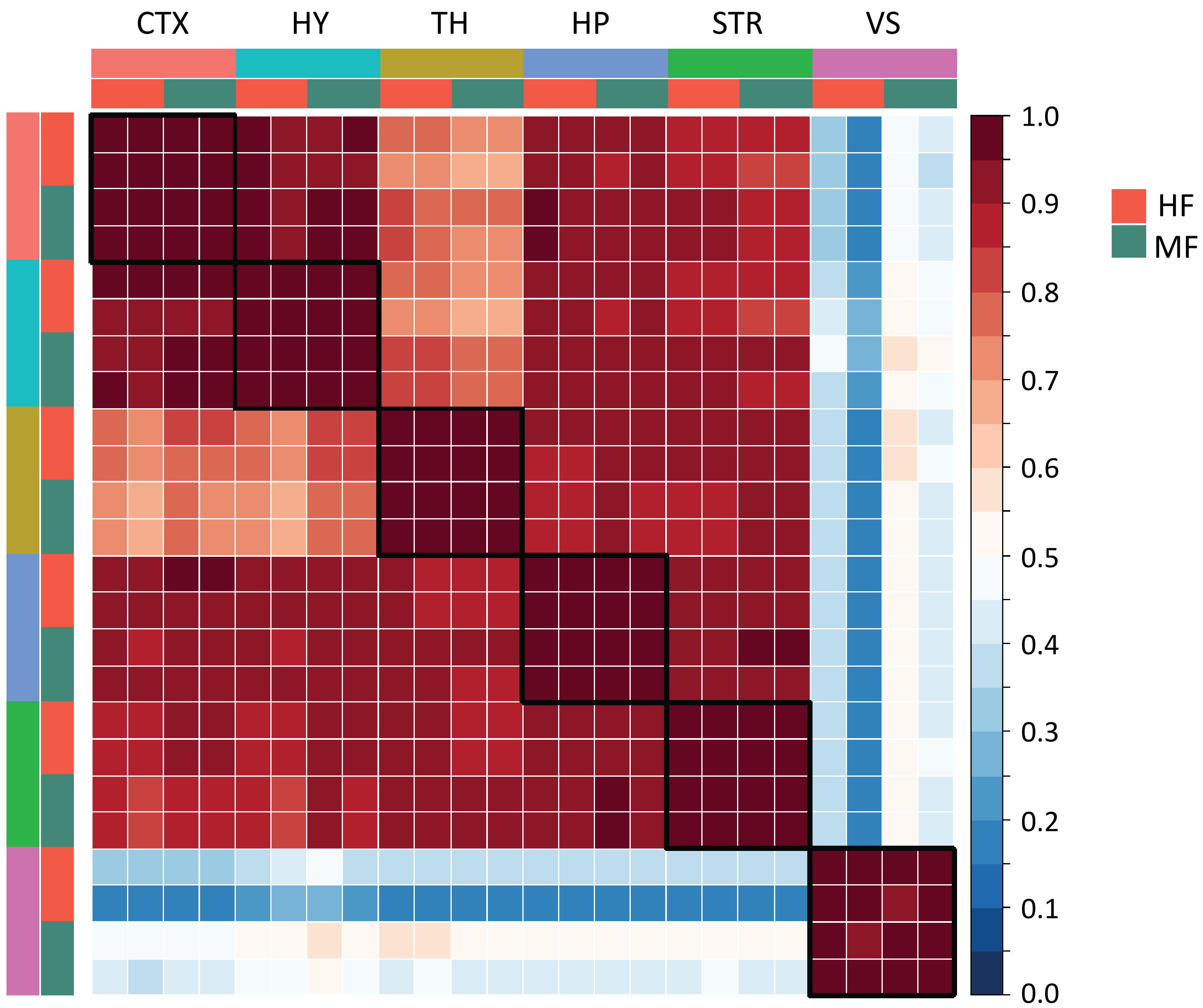

Supplementary Figure S3. Replicate reproducibility. Heatmap showing the correlation of spatial transcriptomic profiles between two biological replicates across six brain regions for both MF and HF groups.

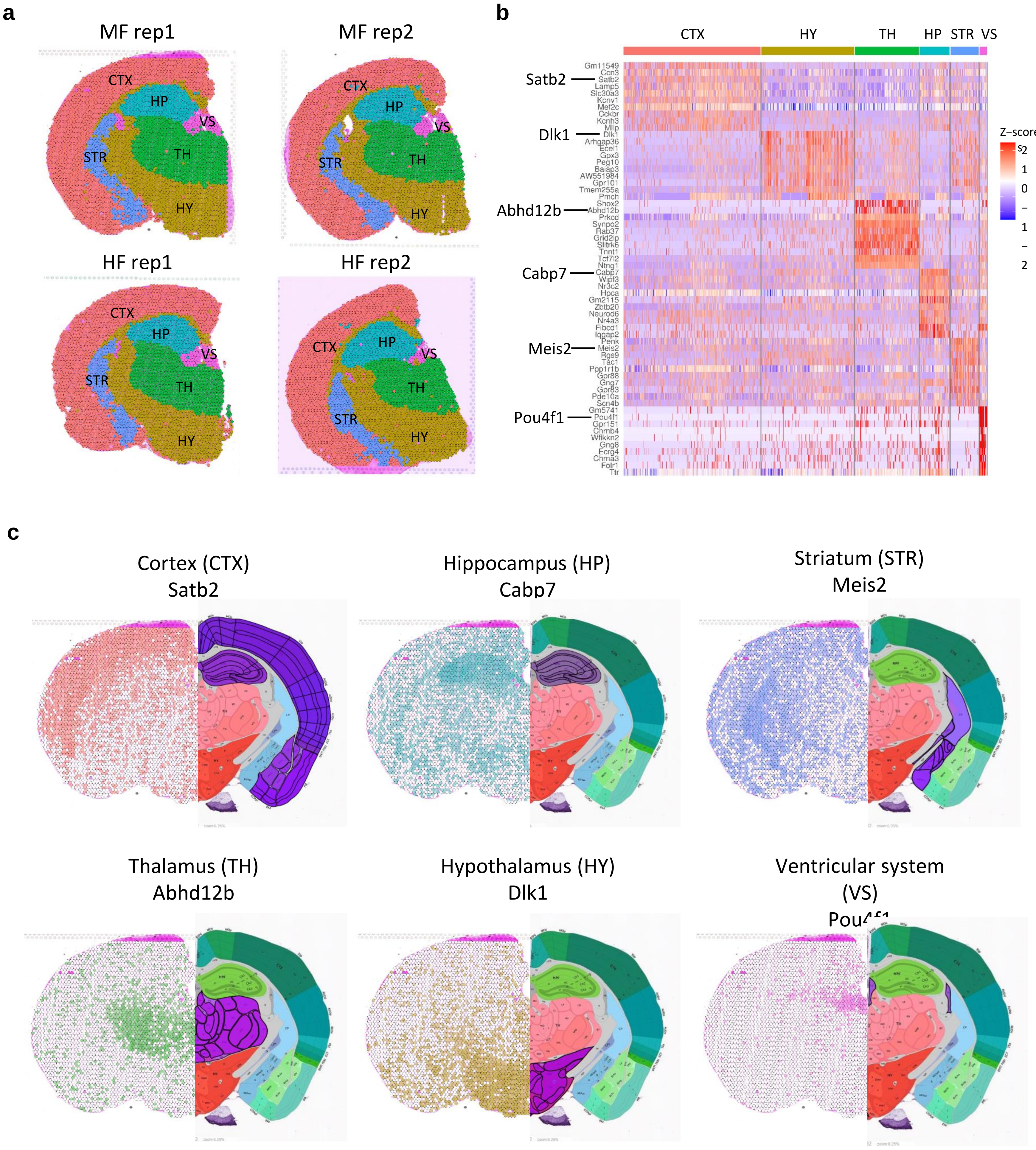

Supplementary Figure S4. FFPE Visium spatial transcriptomics in agreement with anatomical regions. a. Annotation of the six major clusters based on their co-localization with anatomical landmarks from the mouse P56 coronal section #72 of the Allen Mouse Brain Atlas. **b**. Heatmap showing the top 10 marker genes for each brain region. **c**. Spatial density plot of representative marker genes for each brain region (left) and the corresponding anatomical landmarks (right).

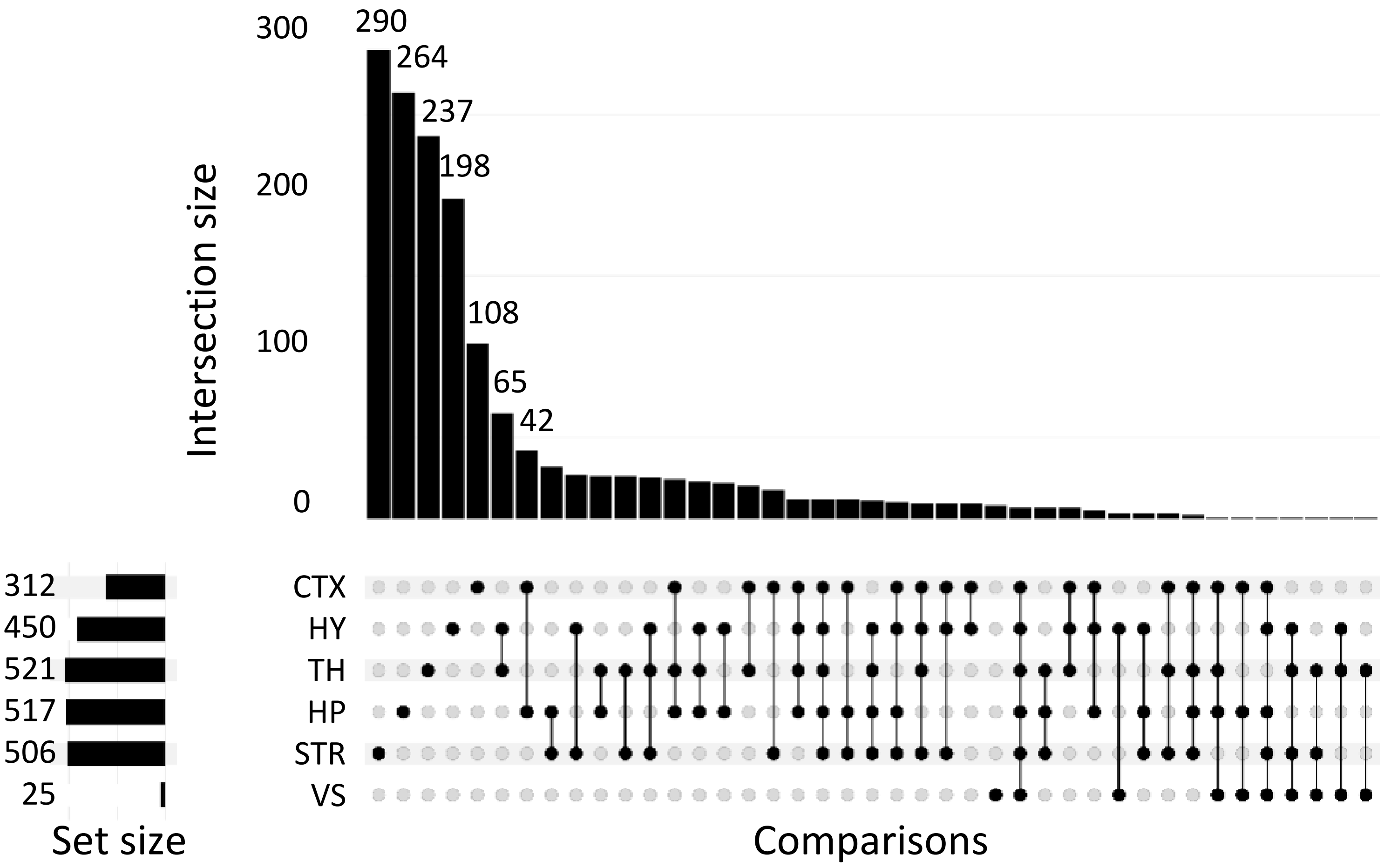

Supplementary Figure S5. Shared and region−specific DEGs among various brain regions. Intersection bar plot showing the number of shared and brain region−specific differentially expressed genes. The horizontal bar on the bottom left side shows the number of DEGs. Different intersection combinations of DEGs are represented by the dot plot. The vertical bar plot shows the number DEGs in the indicated combination of intersection.

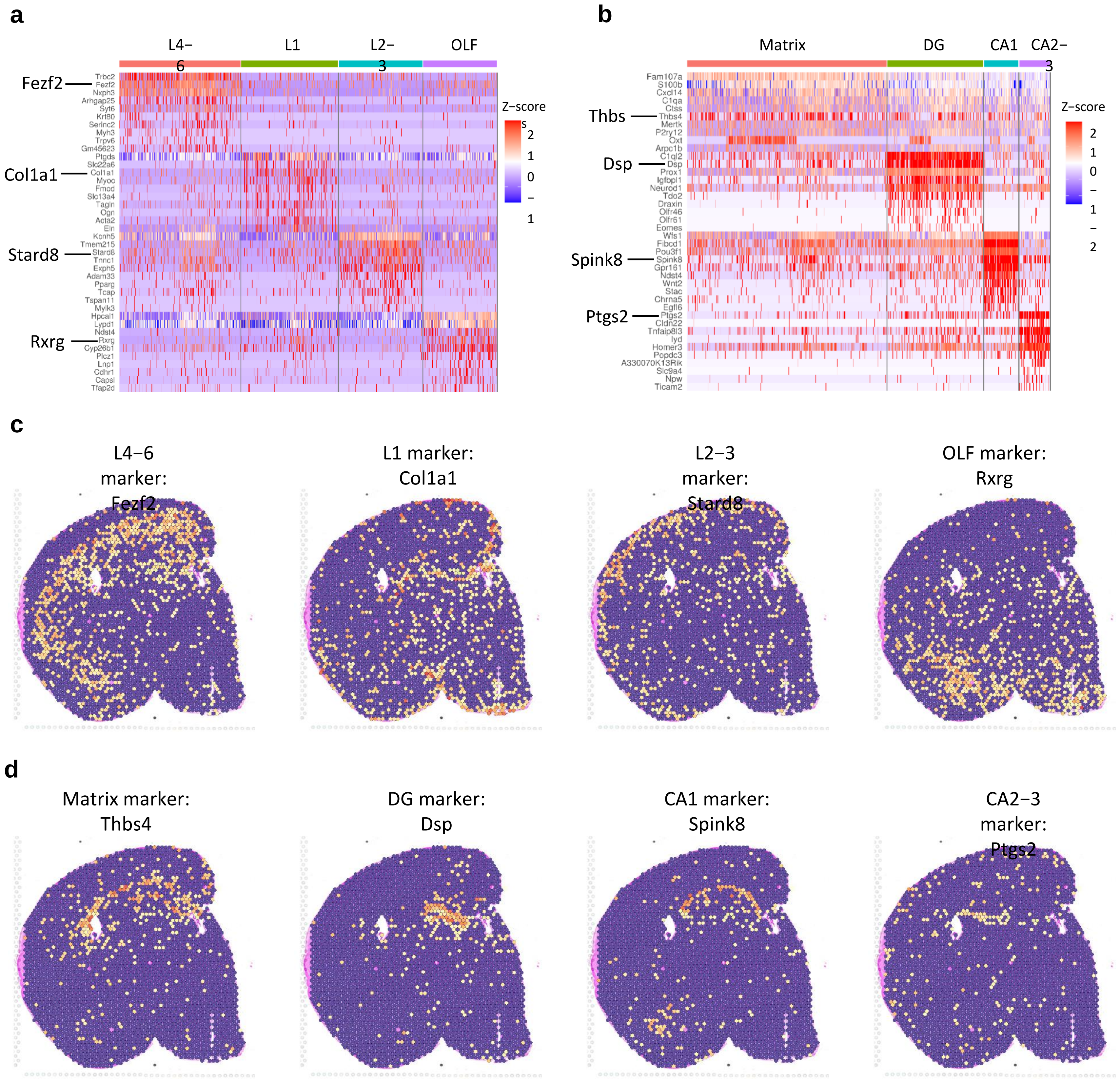

Supplementary Figure S6. FFPE Visium spatial transcriptomics aligns subregions in the cortex and hippocampus. a. Heatmap displaying the top 10 marker genes for each sub−cluster in the cortex region. **b**. Heatmap showing the top 10 marker genes for each sub−cluster in the hippocampus region. **c**. Spatial density plot of representative marker genes for each sub−cluster in the cortex region. **d**. Spatial density plot of representative marker genes for each sub−cluster in the hippocampus region.

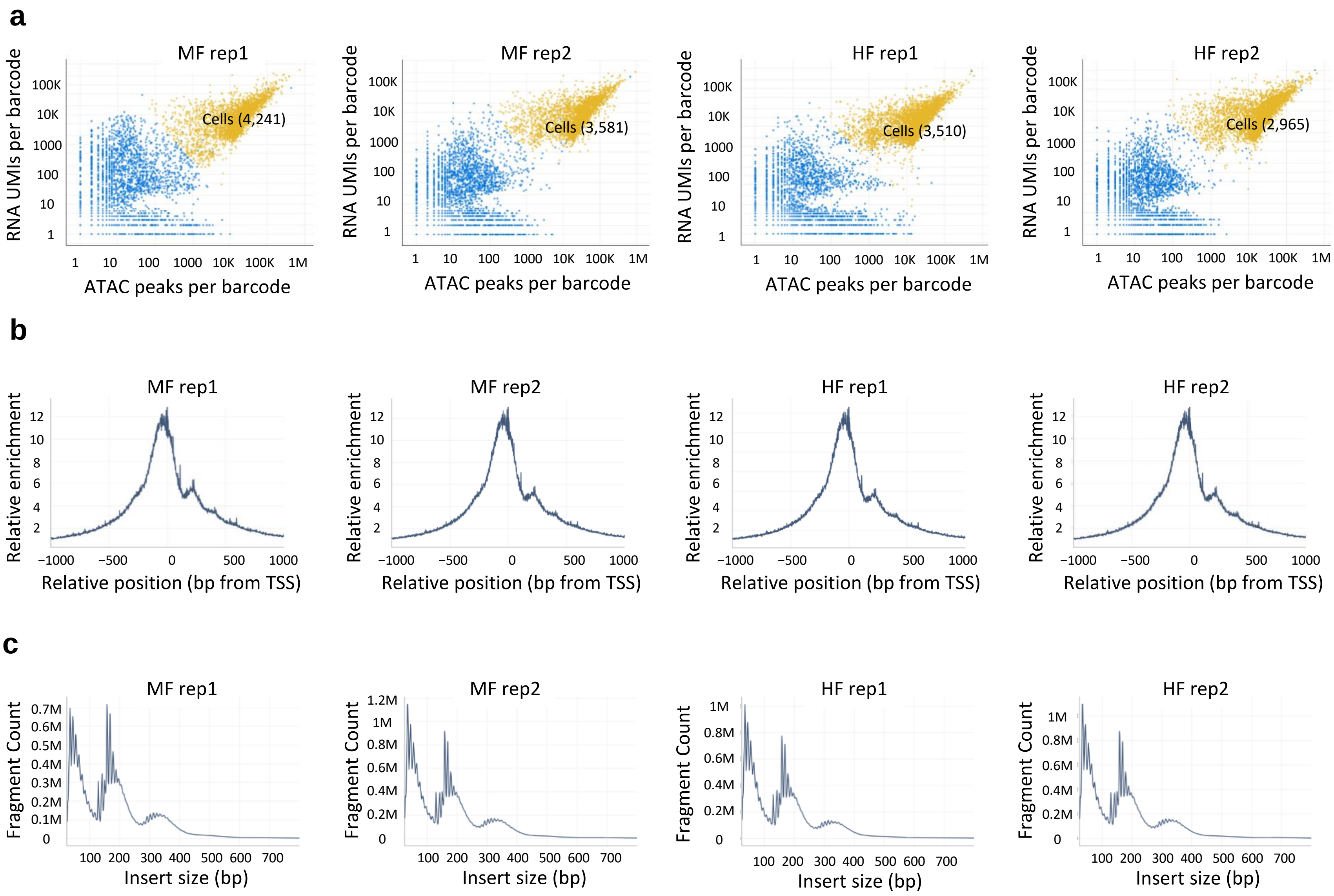

Supplementary Figure S7. Confirmation of the quality of single–nucleus multiome libraries. a. Scatter plot showing the joint cell calling using the cross sensitivities between ATAC transposition events in peaks per barcode and RNA UMIs per barcode. Spots representing cells were highlighted in yellow and the number of cells for each sample was labelled. **b**. Enrichment of ATAC signal around the transcription start sites (TSS) in the four samples. **c**. Distribution of fragment size for nuclei in each sample that passed the quality control threshold.

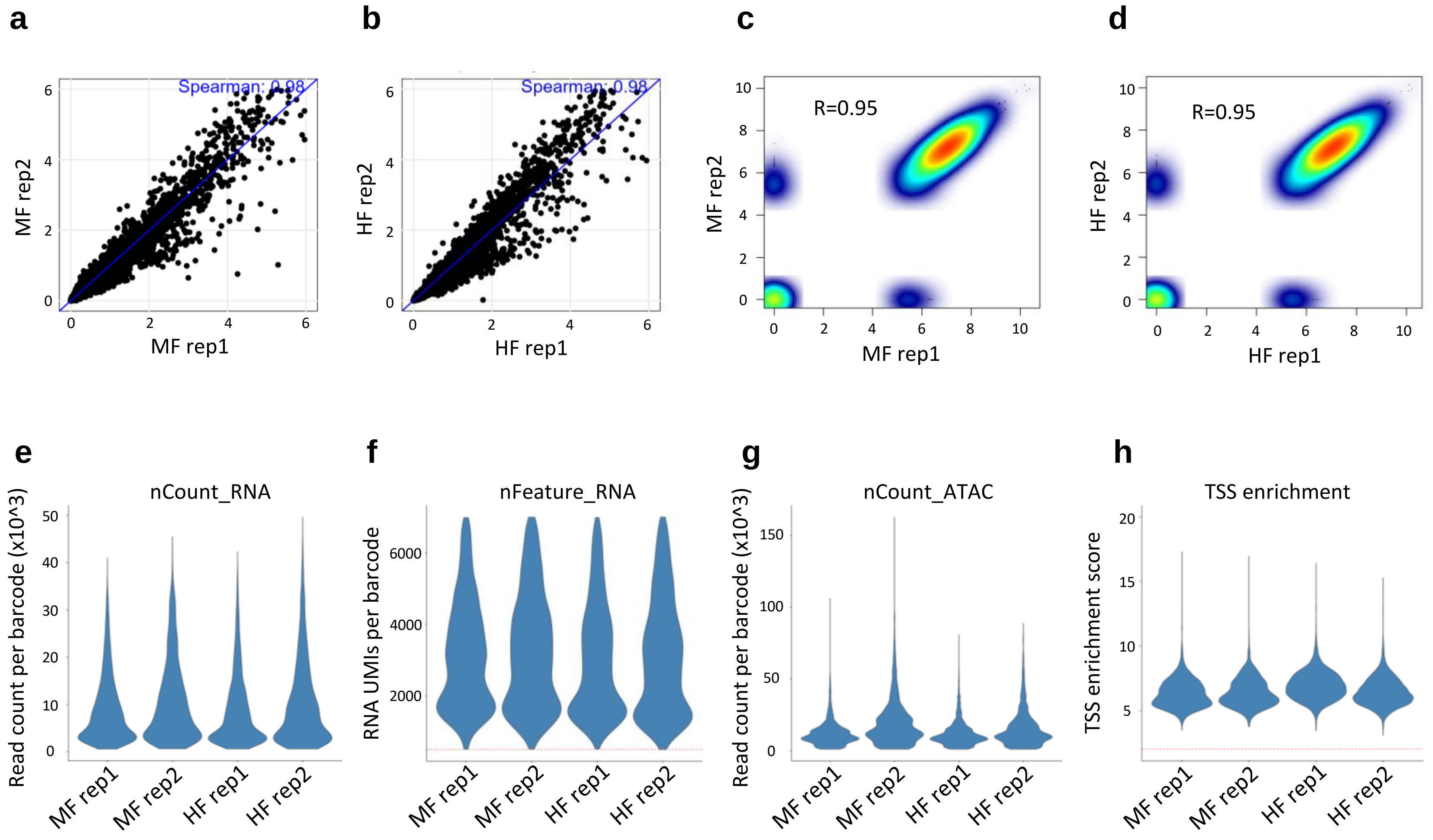

Supplementary Figure S8. Quality control of single−nucleus multiome datasets. a,b. Dot plot showing the correlation of snRNA−seq datasets between two biological replicates, represented by log2(normalized counts) in the MF (**a**) and HF (**b**) groups. **c, d**. Dot plot showing the correlation of snATAC−seq datasets between two biological replicates, represented by log2(RPKM), in the MF (**c**) and HF (**d**) groups. **e**. Violin plot showing the nCount (number of reads) of snRNA−seq datasets. **f**. Violin plot showing the nFeature (number of genes) of snRNA−seq datasets. **g**. Violin plot showing the nCount (number of reads) of snATAC−seq datasets. **h**. Violin plot showing the TSS enrichment scores of snATAC−seq datasets.

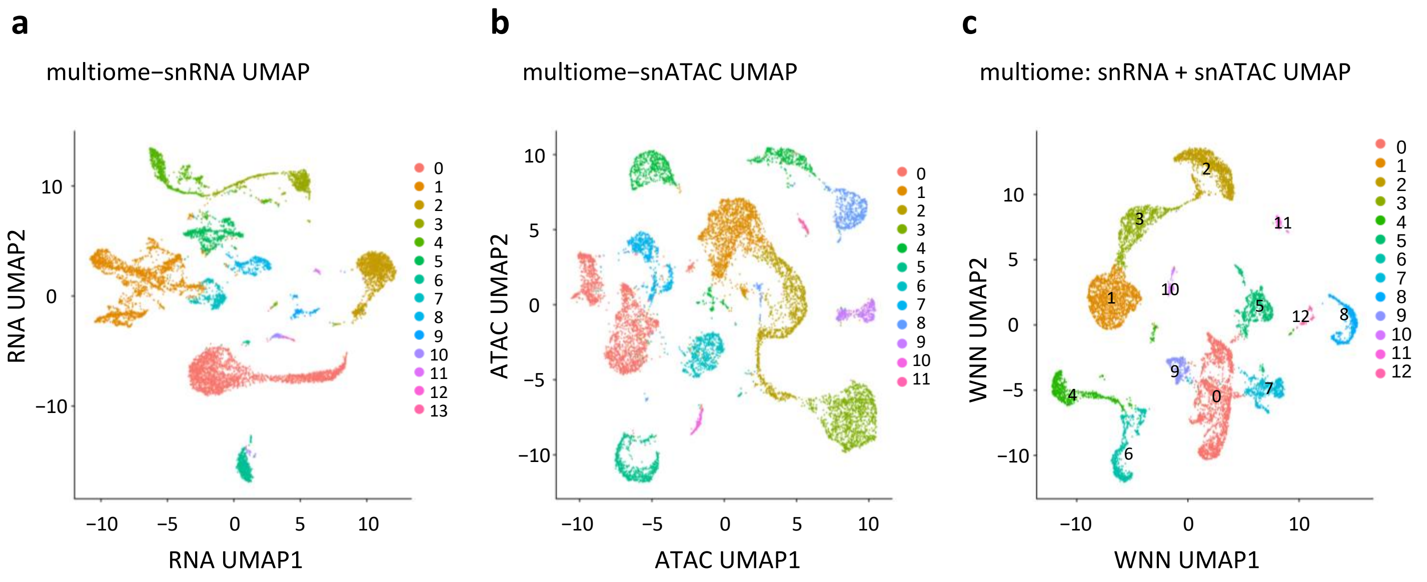

d

Cluster #	Cell type
0<br>1<br>3<br>7<br>9	EXC
5	INH
2	AST
4	OPC
6	ODC
8	MG
10<br>11<br>12	unassigned

Supplementary Figure S9. Clustering of single nucleus multiome datasets. a. UMAP visualization of snRNA−seq datasets. **b**. UMAP visualization of snATAC−seq datasets. **c**. UMAP visualization of combined snRNA−seq and snATAC−seq datasets using WNN approach. Nuclei are colored based on clusters identified in each clustering strategy. **d**. Clusters identified in WNN approach were assigned to major cell types.

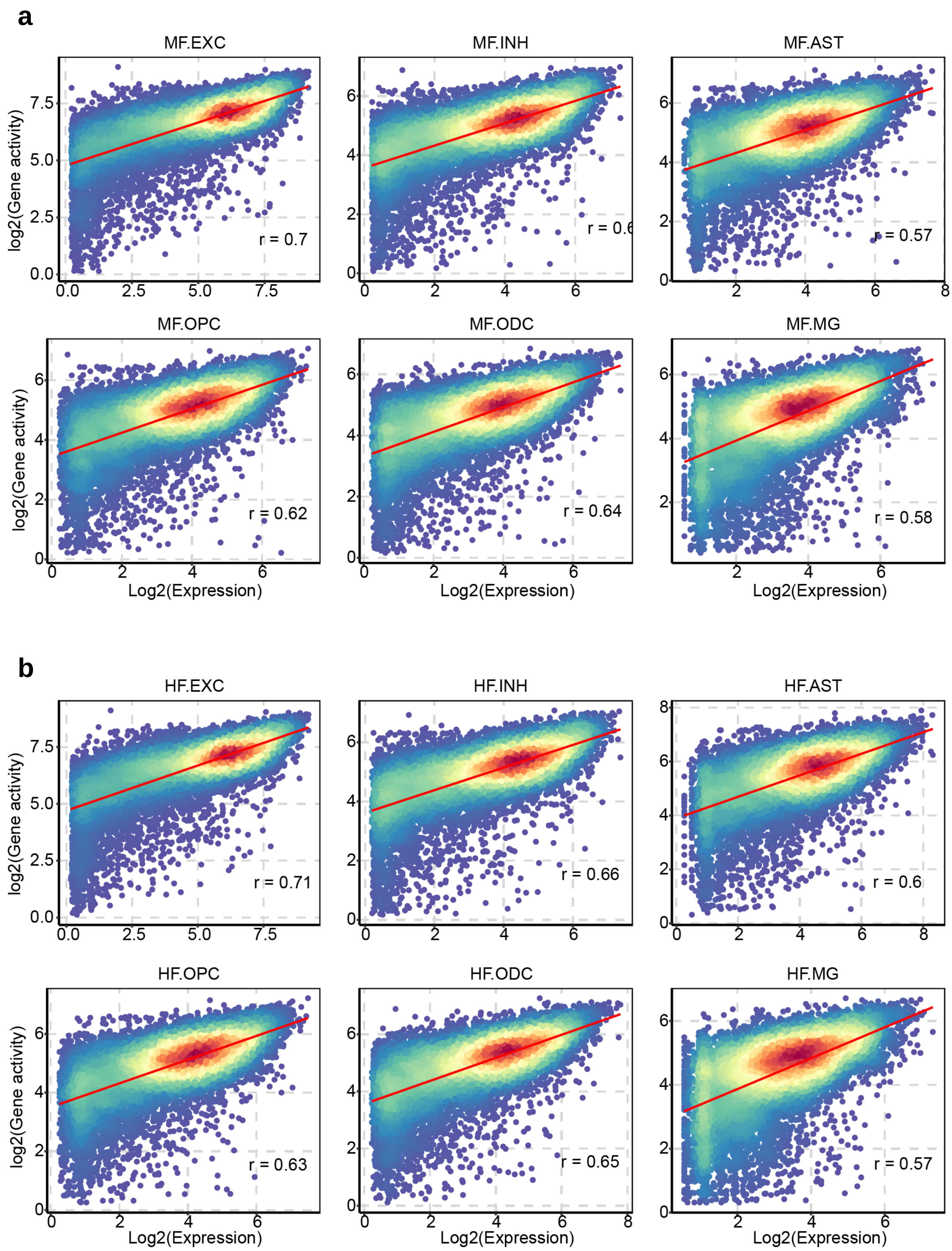

Supplementary Figure S10. Correlation between gene expression and promoter accessibility in major cell types. a. Correlation between gene expression and promoter accessibility for each cell type in the MF group. **b**. Correlation between gene expression and promoter accessibility for each cell type in the HF group.

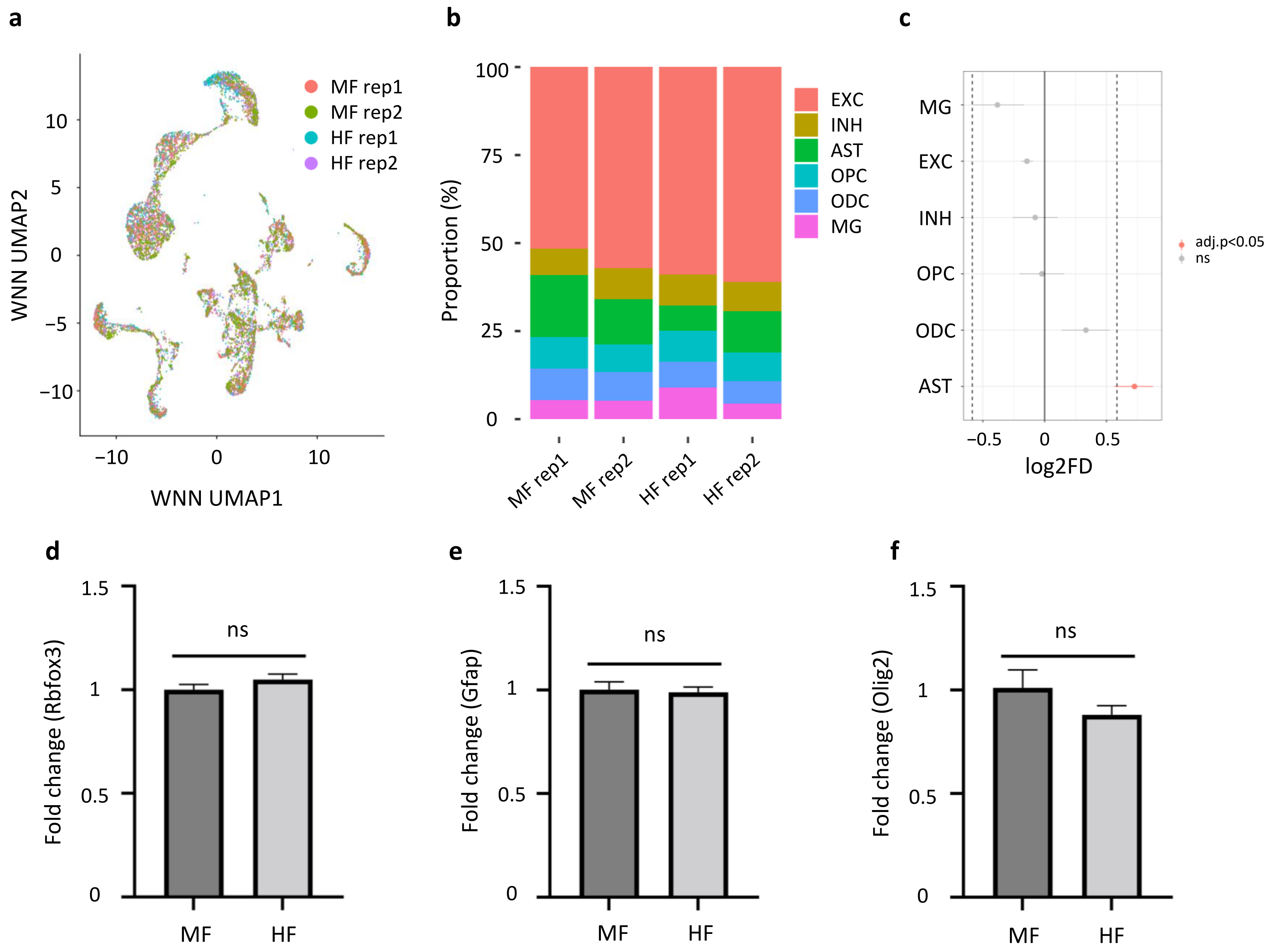

Supplementary Figure S11. Proportion of major cell types identified with the single nucleus multiome. **a**. Integrated UMAP visualization of snRNA−seq and snATAC−seq colored by samples analyzed. **b**. Bar plot showing the Relative percentage of each major cell type in each sample. **c**. Population shift represented by log2 fold change. **d, e, f**. Relative mRNA expression level of Rbfox3 (pan neuronal marker), Gfap (astrocyte marker), and Olig2 (Oligodendrocyte marker) in the hippocampus tissues of P21 male pups. Four biological replicates were included. ns: not significant by t−test.

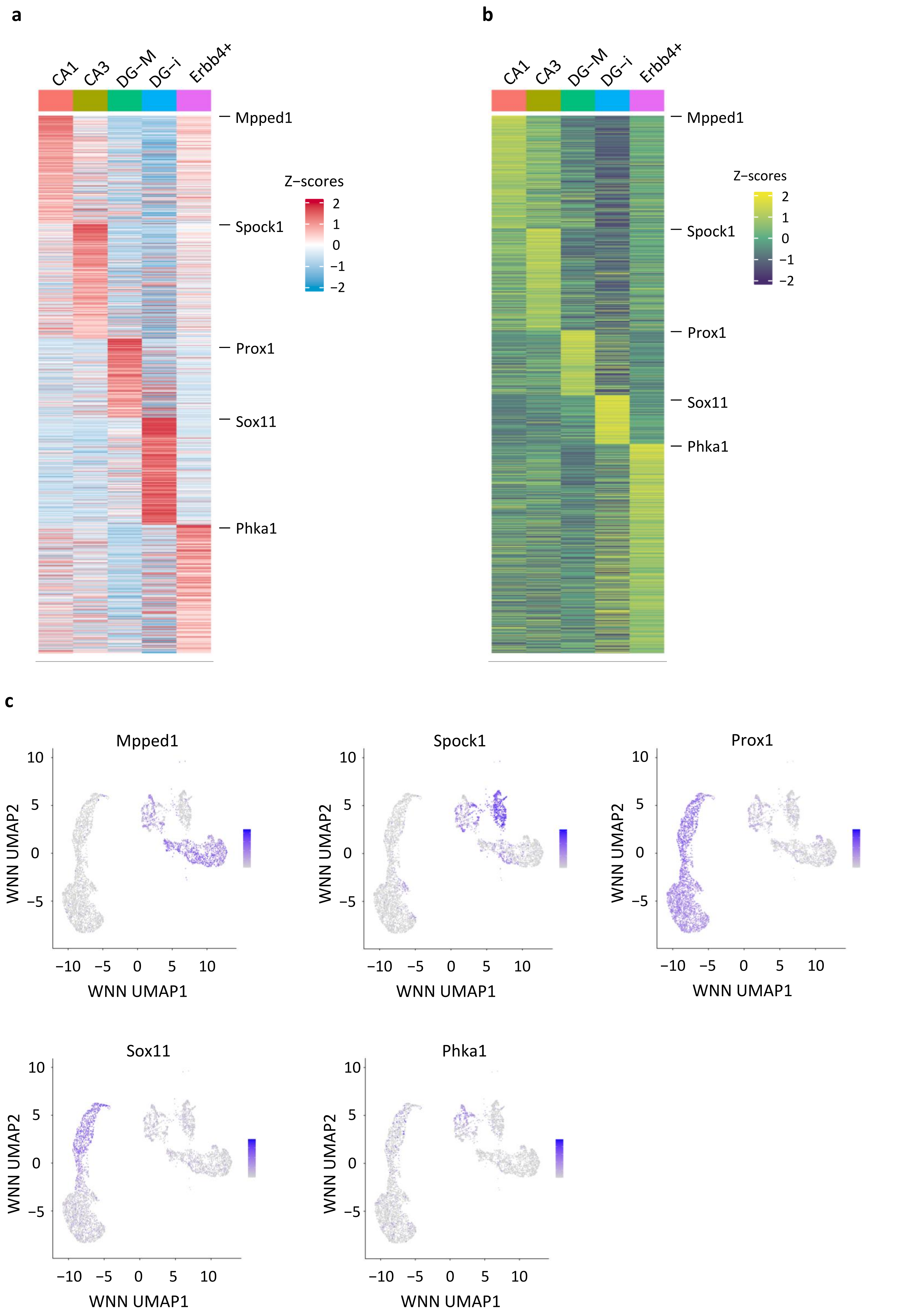

Supplementary Figure S12. Marker genes identified in hippocampal excitatory neuron subtypes. a, b. Row−normalized heatmaps for single nucleus gene expression (a) or gene activity (b) of marker genes specific to excitatory neuron subtypes. **c**. UMAP density plot illustrating the signature genes for each excitatory neuron subtype.

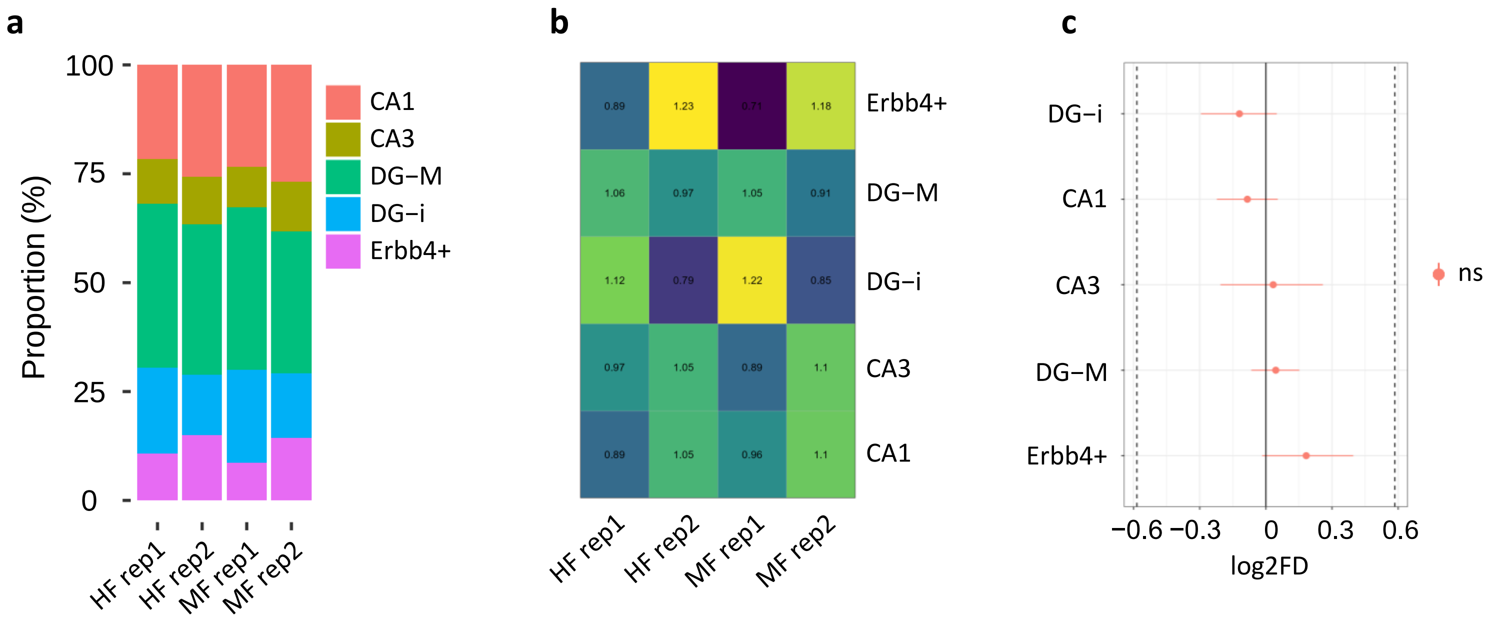

Supplementary Figure S13. Proportion of hippocampal excitatory neuron subtypes identified from the single−nucleus multiome datasets. a. Bar plot showing the proportion of each excitatory neuron subtype in each sample. **b**. Heatmap showing the ratio of observed vs expected of each excitatory neuron subtype. **c**. Population shift represented by log2 fold change. ns: not significant.

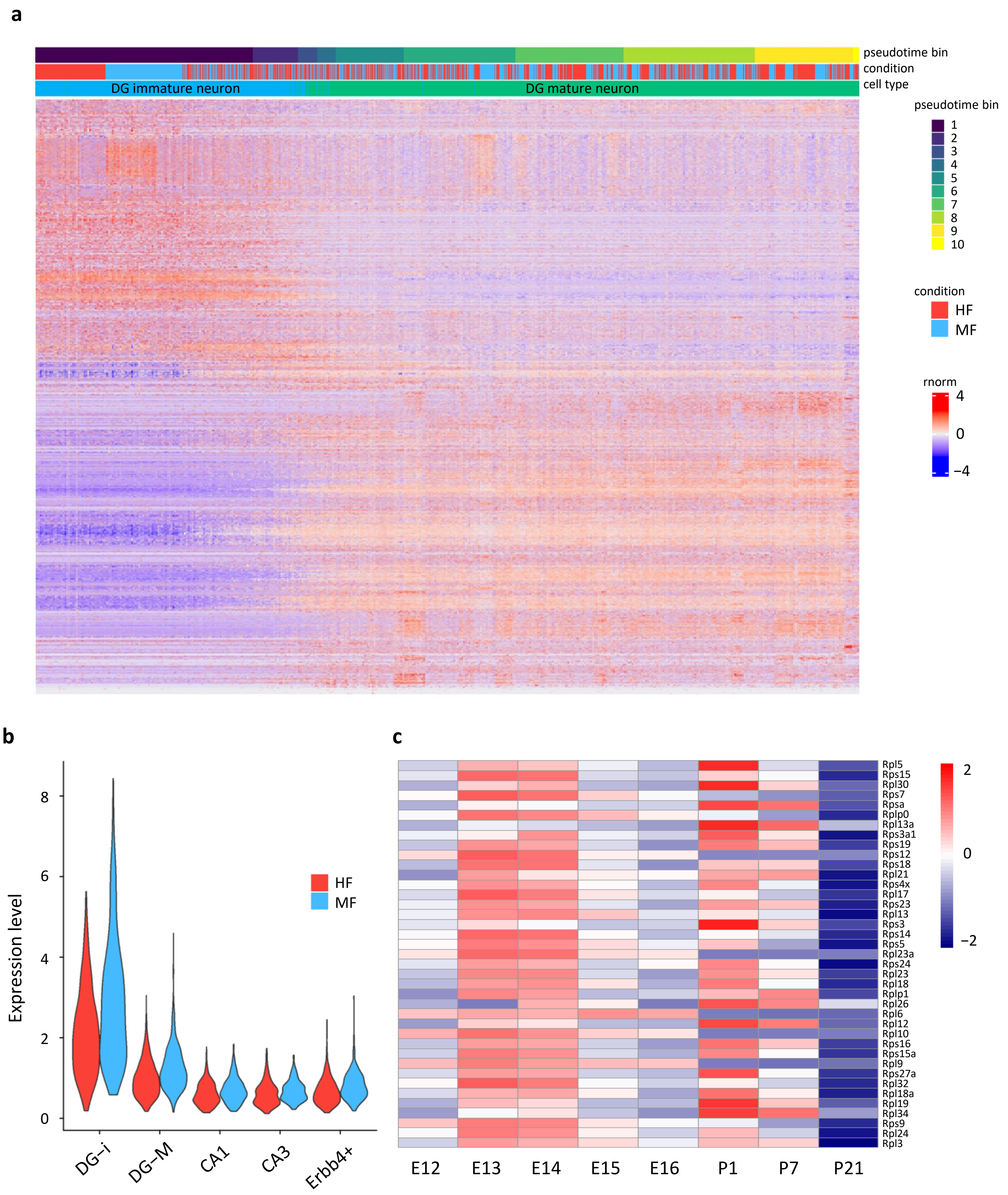

Supplementary Figure S14. Expression profiles of ribosomal protein−coding genes in excitatory neurons. a. Heatmap showing the expression of genes along the trajectory of DG neurons. **b**. Violin plot showing the expression levels of ribosomal protein−coding genes that were both DEGs and trajectory−related in the MF and HF groups across hippocampal excitatory neuron subtypes. **c**. Heatmap showing the expression of trajectory−related ribosomal protein−coding DEGs in excitatory neurons during embryonic and postnatal brain development.

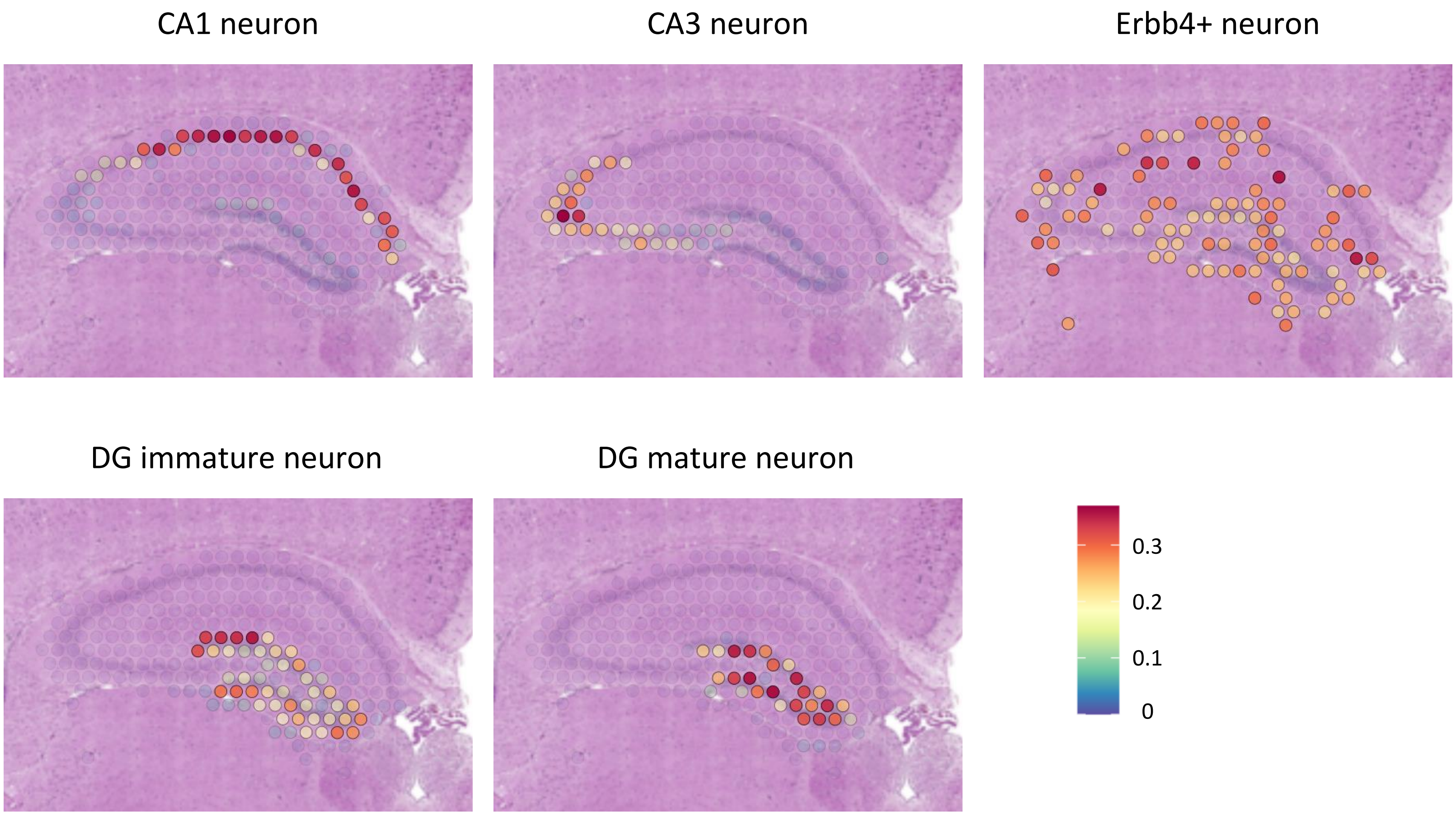

Supplementary Figure S15. Projection of snRNA–seq to spatial transcriptomics. Projection of the hippocampal excitatory neuron subtypes to spatial transcriptomics: CA1 neurons, CA3 neurons, Erbb4+ neurons, DG immature neurons, and DG mature neurons.
